# Effects of a Theory- and Evidence-Based, Motivational Interviewing–Oriented Artificial Intelligence Digital Assistant on Vaccine Attitudes: A Randomized Controlled Trial

**DOI:** 10.2196/72637

**Published:** 2025-08-08

**Authors:** Yan Li, Mengqi Li, Janelle Yorke, Daniel Bressington, Joyce Chung, Yao-Jie Xie, Lin Yang, Mengting He, Tsz-Ching Sun, Angela Y M Leung

**Affiliations:** 1 School of Nursing, The Hong Kong Polytechnic University, 11 Yuk Choi RdHong Kong SAR, 999007, China; 2 Mental Health Research Centre, The Hong Kong Polytechnic University, Hong Kong SAR, China; 3 Research Institute for Intelligent Wearable Systems, The Hong Kong Polytechnic University, Hong Kong SAR, China; 4 Division of Nursing, Midwifery and Social Work, University of Manchester, Manchester, United Kingdom; 5 Faculty of Nursing, Chiang Mai University, Chiang Mai, Thailand; 6 Research Centre for Chinese Medicine Innovation, The Hong Kong Polytechnic University, Hong Kong SAR, China

**Keywords:** attitude, motivational interviewing, artificial intelligence, chatbot, vaccine hesitancy, COVID-19

## Abstract

**Background:**

Attitude-targeted interventions are important approaches for promoting vaccination. Educational approaches alone cannot effectively cultivate positive vaccine attitudes. Artificial intelligence (AI)–driven chatbots and motivational interviewing (MI) techniques show high promise in improving vaccine attitudes and facilitating readiness for vaccination.

**Objective:**

This study aimed to evaluate the effectiveness of a theory and evidence-based, MI-oriented AI digital assistant in improving COVID-19 vaccine attitudes among adults in Hong Kong.

**Methods:**

This 2 parallel-armed randomized controlled trial was conducted from October 2022 to June 2024. Hong Kong adults (N=177) who were vaccine-hesitant were randomly assigned into 2 study groups. The intervention group (n=91) interacted with the AI digital assistant over 5 weeks, including receiving a web-based education program comprising 5 educational modules and communicating with an AI-driven chatbot equipped with MI techniques. The control group (n=86) received WhatsApp (Meta) messages directing them to government websites for COVID-19 vaccine information and knowledge, with the same dosage as the intervention group. Primary outcomes included vaccine hesitancy. Secondary outcomes included vaccine readiness, confidence, trust in government, and health literacy. Outcomes were measured at baseline, postintervention, 3-month, and 6-month follow-up. Focus group interviews were conducted postintervention. Intervention effects were analyzed using the generalized estimating equation model. Interview data were content analyzed.

**Results:**

Decreases in vaccine hesitancy were observed while no statistically significant time-by-group interaction effects were found. The intervention showed significant time-by-group interaction effects on vaccine readiness (*P*=.04), confidence (*P*=.02), and trust in government (*P*=.04). Significant between-group differences with medium effect sizes were identified for vaccine readiness (Cohen *d*=0.52) and trust in government (Cohen *d*=0.54) postintervention, respectively. Increases in vaccine-related health literacy were observed, and a significant time effect was found (*P*=.01). In total, three categories were summarized from interview data: (1) improved vaccine literacy, confidence, and trust in government; (2) hesitancy varied while readiness improved; and (3) facilitators, barriers, and recommendations of modifications on the intervention.

**Conclusions:**

The intervention indicated promising yet significant effects on vaccine readiness while the effects on vaccine hesitancy require further confirmation. The qualitative findings; however, further consolidate the significant effects on participants’ attitudes toward vaccines. The findings provide novel evidence to encourage the adoption and refinement of a MI-oriented AI digital assistant in vaccine promotion.

## Introduction

Vaccination is one of the most cost-effective public health interventions against infectious diseases worldwide. According to the World Health Organization (WHO), vaccination prevents 3.5‐5 million deaths annually from diseases like diphtheria, tetanus, and inﬂuenza [1]. Data estimates from 185 countries and regions suggest that COVID-19 vaccines alone prevented over 20 million deaths globally within the first year of their deployment, underscoring the significant impact of widespread vaccination efforts [2]. Vaccination programs also provide substantial economic benefits by increasing quality-adjusted life years and reducing health care costs in the long run [3]. However, individuals’ attitudes toward vaccines, including vaccine hesitancy and low levels of confidence and readiness, pose a significant threat to global immunization efforts and public health [4]. Recent studies indicate that global vaccine hesitancy remains a significant issue worldwide, with hesitancy rates ranging from 33.3% to 85% reported in 13 countries, representing approximately half of the global population [5]. The persistence of negative vaccine attitudes globally highlights the necessity and importance of developing effective interventions to address this critical public health issue [6,7].

Attitude-targeted interventions by modifying attitudinal factors have been prioritized in vaccine promotion programs, with particular efforts on reducing hesitancy and facilitating individuals’ readiness for vaccination [8]. Vaccine hesitancy is significantly associated with reduced readiness for vaccination and is influenced by multifaceted factors [9]. The Vaccine Hesitancy Determinants Matrix Model, developed by the WHO Strategic Advisory Group of Experts on Immunization, systematically identifies vaccine and vaccination-specific, individual and group, and contextual inﬂuences on vaccine hesitancy [10]. This model provides solid theoretical underpinnings for studies exploring the significant influencing factors of vaccine hesitancy, thereby guiding the development of effective interventions. Previous evidence has demonstrated significant modifiable factors influencing vaccine hesitancy, including vaccine confidence (vaccine-specific influences such as concerns about vaccine safety and efficacy) [11], personal health literacy (individual influences) [12], and trust in government (contextual influences) [13]. These factors are recommended to be included in attitude-targeted interventions for decreasing vaccine hesitancy and improving the uptake of vaccines [14]. Educational campaigns have been widely introduced in vaccine promotion programs worldwide to improve individuals’ knowledge and confidence in vaccine safety and efficacy, as well as their trust in government-provided vaccine information and policies [15-17]. These initiatives primarily involved educational intervention delivered through booklets, phone calls, and Microsoft PowerPoint presentations. However, relying exclusively on didactic education is inadequate for effectively fostering positive attitudes toward vaccines and encouraging behavior change regarding vaccination [18].

Motivational interviewing (MI), a client-centered counseling method, has proven highly effective in addressing ambivalence and enhancing motivation to change [19,20]. MI has shown promise in promoting vaccination by fostering a supportive dialogue that addresses individuals’ ambivalence toward vaccines [21]. MI techniques integrated with educational information delivered by health professionals have shown positive effects in reducing hesitancy toward vaccines, such as the human papillomavirus vaccine [22] and postpartum infant vaccines [23]. The personalized and empathetic approach of MI can help address specific concerns and misinformation, making it a valuable tool in reducing COVID-19 vaccine hesitancy [24]. In addition, artificial intelligence (AI)–driven chatbots have emerged as valuable tools in promoting vaccination by providing timely, personalized information for a large number of users simultaneously and conserving health care resources [25]. Chatbots have been introduced in COVID-19 vaccine promotion in Hong Kong, Thailand, Singapore, and France, and have demonstrated significant effectiveness in enhancing vaccine confidence and reducing vaccine hesitancy through conversations compared with passive learning educational materials [26,27]. Furthermore, chatbots can be empathetic by integrating with therapeutic MI techniques and have shown significant effects in reflecting on ambivalence and supporting self-efficacy, contributing to increased motivation and commitment to quitting smoking [28]. However, the integration of MI and AI chatbots has not yet been tested to improve vaccine attitudes and address vaccine hesitancy.

Negative vaccine attitudes are significant global health concerns and are particularly prevalent in high-income countries or regions [5]. In Hong Kong, negative vaccine attitudes pose a considerable barrier to public health efforts, with over 30% of the population remaining hesitant toward COVID-19 vaccines despite extensive efforts by the government and health care system to promote vaccination [29]. It is important to bridge the gap between vaccination campaigns and coverage by reducing vaccine hesitancy through accessible and effective, attitude-targeted interventions [30]. MI techniques and AI chatbots hold high promise in disseminating vaccine information and fostering positive attitudes and motivation to get vaccinated. This study aimed to evaluate the effectiveness of a theory and evidence-based, MI-oriented AI digital assistant in improving COVID-19 vaccine attitudes among adults in Hong Kong using a randomized controlled trial. The findings can provide high-quality and novel evidence to adopt effective vaccine promotion approaches for coping with COVID-19 and other vaccine-preventable diseases.

## Methods

### Study Design

The study adopted a 2 parallel-armed randomized controlled trial design with repeated measures at baseline (T0), postintervention (T1), 3-month (T2), and 6-month follow-ups (T3). Qualitative interviews with focus groups involving participants in the intervention group were conducted postintervention to explore participants’ views about the intervention’s acceptance, strengths, and limitations, and suggested improvements.

### Ethical Considerations

This study was approved by the Institutional Review Board of the Hong Kong Polytechnic University (HSEARS20221031003). Informed consent (Multimedia Appendix 1) was obtained from all participants before their participation. Participants were assured that their responses would remain confidential and that all data would be anonymized and stored securely. Only the research team had access to the data, and all identifying information was removed before analysis. The trial protocol was prospectively registered in ClinicalTrials.gov. There were no deviations from the prospectively registered protocol.

### Participants and Sample Size

The trial was conducted in Hong Kong between October 2022 and June 2024. Recruitment involved collaborating with local district health centers to deliver email, call, or poster invitations and social media promotion (ie, Twitter [rebranded as X] and Facebook [Meta]). Participants were included if they (1) are Hong Kong residents aged 18 years or more, (2) are vaccine hesitant (not taking COVID-19 vaccines or receiving involuntary COVID-19 vaccines such as receiving vaccines due to government, school, or employer mandate), (3) have internet access, and (4) are able to read Chinese or English. Participants were excluded if they (1) had medically diagnosed cognitive impairments or mental disorders, or (2) were currently engaged in other COVID-19 vaccine promotion programs. The sample size was determined using the GLIMMPSE 3.0.0 software (University of Colorado Denver). The calculation, based on estimated correlations among repeated measures (0.6 for T0-T1, 0.5 for T0-T2, and 0.4 for T0-T3), a statistical power of 80%, and an attrition rate of 20%, indicated a total of 168 participants (84 per group) would be the minimum required for the study. A purposive sampling by key informants in the intervention group (containing those who improved most and least on vaccine hesitancy postintervention) was conducted for focus-group interviews, until information saturation was reached as no relevant new codes were found in data.

### Randomization and Masking

Eligible participants were randomly assigned to 1 study group (1:1) using a stratified randomization based on the age groups (ie, 18‐29 y, 30‐44 y, 45‐59 y, and 60 y or older). To ensure allocation concealment, random assignment was performed by an external administrator using a web-based randomization service (sealed envelope website). Upon written consent and the completion of the baseline assessment, the external administrator inputted participant numbers into the Sealed Envelope system to determine group assignment for each age group. The group allocation was subsequently disclosed to the research team and participants. Due to the nature of the intervention, participants could not be masked but were reminded to keep it confidential to prevent contamination. Furthermore, the intervention and control groups interacted within separate platforms to receive their respective treatments. Only the intervention group was provided with accounts to access the AI digital assistant for completing the intervention. The data collector and data analyst were blinded to group assignment.

### Intervention

**Figure 1.**
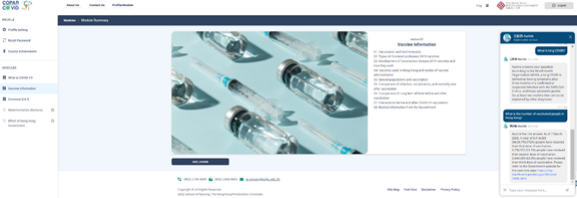
The interface of the artificial intelligence digital assistant.

The development of the AI digital assistant was theoretically underpinned by the Vaccine Hesitancy Determinants Matrix Model and evidence-supported by qualitative interviews with Hong Kong adults. The AI digital assistant was validated by an expert review process and pilot-tested by users. The details of the intervention development, validation, and pilot test have been reported [31]. The AI digital assistant incorporates a web-based educational program with a MI-oriented, AI-driven chatbot “Auricle” (Digital Psychosocial Health Research Group, School of Nursing, The Hong Kong Polytechnic University). The web-based educational program consists of five modules designed to address vaccine hesitancy (Multimedia Appendix 2): (1) basic knowledge of COVID-19, (2) basic knowledge of COVID-19 vaccines, (3) common questions about COVID-19 vaccines, (4) myths about COVID-19 vaccines, and (5) efforts by the Hong Kong government. Each module was embedded with an AI-driven chatbot, which provided MI-oriented dialogues, encouraging users to reflect on the educational content and initiating their motivation to vaccinate. The 4 processes (ie, engaging, focusing, evoking, and planning) and 4 principles (ie, expressing empathy, developing discrepancy, rolling with resistance, and supporting self-efficacy) were included in MI-oriented dialogues. A logical algorithm was used to structure the MI-oriented dialogues into the AI-driven chatbot system by professionals in computer science. The AI-driven chatbot was powered by natural language processing to analyze user input and provide real-time and personalized conversations. “Auricle” also provided assessments of vaccine importance, confidence, and readiness with single-item questions rating from 0 to 10 at the end of dialogues [31]. Participants progressed through 1 module per week for 1 hour over a 5-week period. After self-learning each module’s educational content, scoring over 80% on multiple-choice questions, and finishing dialogues with “Auricle” within a week, a new module was released for learning in the next week. Figure 1 displays the interface of the AI digital assistant.

Participants in the control group received weekly WhatsApp (Meta) messages directing them to government websites relevant to basic knowledge, common questions, myths about the COVID-19 vaccine, and government efforts over 5 weeks. They were also required to complete multiple-choice questions each week, as the participants in the intervention group did. Adherence was evaluated based on their completion of multiple-choice questions.

### Intervention Fidelity and Safety

Before the commencement of the intervention, participants in the intervention group received an electronic user manual. The manual instructed participants to use the AI digital assistant with detailed procedures, including guidance for the initial account registration, self-learning with the web-based educational modules, chatting with “Auricle,” and frequent questions and answers. An automatic email was sent to participants to remind them to complete the module learning weekly. Participants who completed at least 2 out of 5 modules were considered completers and were included in data analysis. A research assistant weekly checked the dashboard of the AI digital assistant to follow up on the intervention progress on module content learning, multiple choice practice, and the interaction with “Auricle.” The research assistant also sent messages and made calls to participants using WhatsApp to encourage their engagement in the intervention. Any adverse events occurring during the study were handled cautiously and recorded.

### Measures

#### Sociodemographics and Vaccination History

Sociodemographics and vaccination history include age, gender, monthly household income, employment, educational level, health status, chronic illness history, COVID-19 infection history, COVID-19 vaccination history, and side effect experience.

#### Primary Outcome

Vaccine hesitancy was measured using the 10-item Adult Vaccine Hesitancy Scale. The scale was rated on a 5-point Likert scale ranging from “strongly disagree” to “strongly agree.” The total scores range from 10 to 50, with higher scores indicating lower hesitancy. The scale demonstrated acceptable reliability (Cronbach α=0.73) in the Chinese population [32].

#### Secondary Outcomes

Vaccine readiness was measured by a commonly used single-item question “How ready are you to receive a COVID-19 vaccine?” with responses on an 11-point scale from 0 (not ready at all) to 10 (highly ready) [33].

Vaccine confidence was measured with the 4-item Vaccine Confidence Index, with total scores ranging from 4 to 20 and higher scores indicating greater confidence. The index showed satisfactory reliability (Cronbach α=0.83) in the Chinese population [34].

Trust in government was measured using 1 commonly used item “Please rate your level of trust in the HK government to provide the best possible information about COVID-19 vaccine from 0 (not trust at all) to 100 (completely trust)” [34].

Vaccine-related health literacy was assessed using 4 items from the disease prevention domain of the Chinese version of the European Health Literacy Survey Questionnaire. These items assessed participants’ ability to find, understand, judge, and decide on vaccinations. Responses were rated on a 4-point Likert scale, ranging from “very easy” (4) to “very difficult” (1), with an excellent internal consistency (Cronbach α=0.91) [35].

#### Acceptability

The acceptability of the intervention was indicated by (1) adherence rate (ie, the number of intervention sessions attended divided by the total number of required sessions); (2) satisfaction (participants’ satisfaction with the intervention was assessed using a general scale, the 8-item Client Satisfaction Questionnaire rated on a 4-point Likert scale [1=lowest, 4=highest] [36], the total score ranges from 8 to 32 where higher scores indicate greater satisfaction); and (3) qualitative feedback (the acceptability of the intervention was also evaluated by focus-group interviews with a purposive sample of participants who improved most and least on vaccine hesitancy post the intervention. Participants’ perspectives about the strengths, limitations, and suggested improvements of the interventions were collected.

### Data Analysis

Quantitative data were numerically coded and analyzed using IBM SPSS version 26. Intention-to-treat analysis was conducted using the last observation carried forward for missing data. Descriptive statistics were used to summarize the data. Randomized testing between 2 study groups at baseline was conducted for categorical variables using the chi-square test, while continuous variables were analyzed using independent *t* tests or Mann-Whitney *U* tests. Intervention effectiveness was assessed using the generalized estimating equation model, controlling for covariates identified by the randomized testing. The model evaluated group, time, and interaction effects, with post hoc comparisons performed to identify significant between-group differences using estimated marginal means [37]. Effect sizes for between-group comparisons were calculated using Cohen *d* [38]. The 30% top outliers of the outcomes were removed in data analysis [39]. A sensitivity analysis was conducted, and the generalized estimating equation model results using complete case analysis are provided in the Multimedia Appendix 3 supporting the robustness of the findings. Tape-recorded interviews were transcribed into Cantonese by 1 researcher (T-CS) and cross-checked by the other researcher (ML). Qualitative data generated from interview transcripts were content-analyzed independently by 2 researchers (T-CS and ML) with training in qualitative content analysis following the process of open coding, creating categories, and abstraction [40]. Any discrepancy in coding and categories was discussed and finally confirmed by the research team and explained with verbatim data.

## Results

### Recruitment and Participant Flow

**Figure 2.**
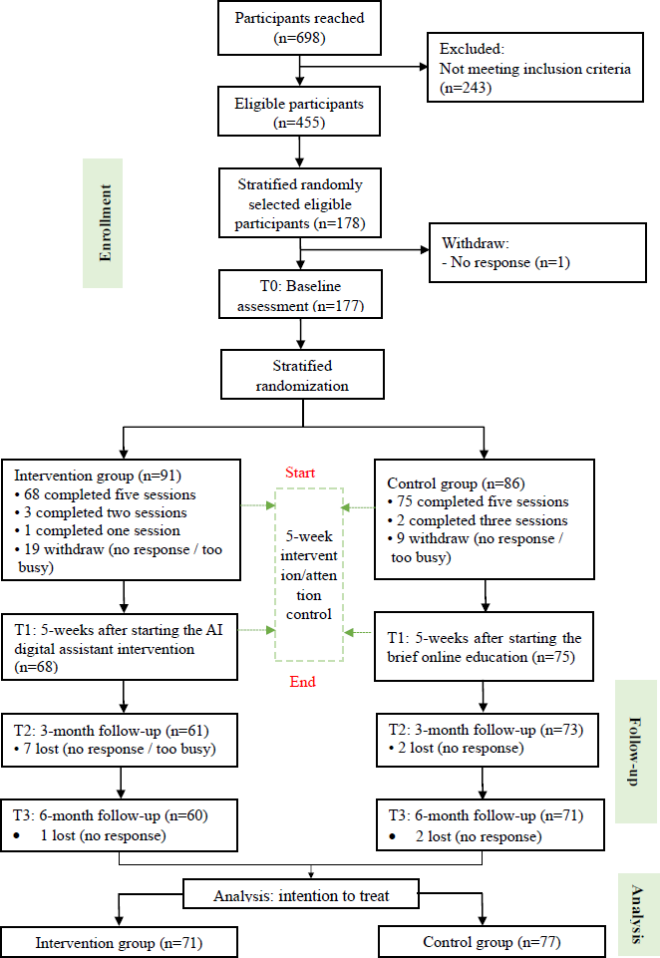
Participant flow chart. AI: artificial intelligence.

The participants’ recruitment occurred from October 2022 to May 2023, with follow-up concluding in June 2024. The study flowchart in Figure 2 adheres to the CONSORT (Consolidated Standards of Reporting Trials) guidelines (the CONSORT-EHEALTH [Consolidated Standards of Reporting Trials of Electronic and Mobile Health Applications and Online Telehealth] checklist is provided in Checklist 1). A total of 178 participants were selected from 455 eligible participants using stratified randomization based on 4 age groups. However, 1 of 178 participants lost contact. Finally, 177 eligible participants were allocated using stratified randomization based on 4 age groups, with 91 assigned to the intervention group and 86 to the control group. Table 1 presents the detailed number of participants in each age group. The adherence rate of completing required sessions was 76.3% and 88.6% for the intervention and control group, respectively. No direct intervention-related injuries or adverse effects were reported.

**Table 1.** Participant characteristics and outcomes at baseline.

Characteristics	Total (n=177)	Intervention (n=91)	Control (n=86)	*t* test (*df*), *z* score, or Chi-square (*df*)	*P* value
Age, mean (SD)	43.38 (16.42)	43.48 (16.14)	43.28 (16.81)	0.083 (175)^a^	.93
Age group (y), n (%)				0.163 (3)^b^	.98
18‐29	46 (26)	23 (25)	23 (27)		
30‐44	46 (26)	23 (25)	23 (27)		
45‐59	43 (24)	23 (25)	20 (23)		
60 and above	42 (24)	22 (25)	20 (23)		
Sex, n (%)				0.126 (1)^b^	.72
Male	62 (35)	33 (36)	29 (34)		
Female	115 (65)	58 (64)	57 (66)		
Educational level, n (%)				1.147 (2)^b^	.56
No education	2 (1)	1 (1)	1 (1)		
Secondary or below	61 (35)	28 (31)	33 (38)		
College or above	114 (64)	62 (68)	52 (61)		
Employment, n (%)				0.414 (1)^b^	.52
Employed	97 (55)	52 (57)	45 (52)		
Unemployed	80 (45)	39 (43)	41 (48)		
Monthly household income^c^, n (%)				3.122 (4)^b^	.54
$20,000 or below	56 (32)	28 (31)	28 (33)		
$20,000-$39,999	51 (29)	23 (25)	28 (33)		
$40,000-$59,999	38 (21)	24 (26)	14 (16)		
$60,000-$79,999	21 (12)	11 (12)	10 (11)		
$80,000 or above	11 (6)	5 (6)	6 (7)		
Chronic illness, n (%)				0.004 (1)^b^	.95
Yes	25 (14)	13 (14)	12 (14)		
No	152 (86)	78 (86)	74 (86)		
Health status, n (%)				1.783 (3)^b^	.62
Very good	13 (7)	9 (10)	4 (5)		
Good	76 (43)	38 (42)	38 (44)		
Fair	80 (45)	40 (44)	40 (46)		
Bad	8 (5)	4 (4)	4 (5)		
Infection history, n (%)				1.249 (3)^b^	.74
Yes	121 (68)	62 (68)	59 (67)		
Probably	10 (6)	4 (4)	6 (7)		
No	43 (24)	24 (27)	19 (22)		
Prefer not to say	3 (2)	1 (1)	2 (2)		
Side effect experience, n (%)				1.354 (2)^b^	.51
No	43 (25)	23 (26)	20 (24)		
Mild to moderate	110 (64)	54 (61)	56 (68)		
Moderate to vigorous	19 (11)	12 (13)	7 (8)		
Current Vaccine Does, n (%)				0.446 (3)^b^	.93
Zero dose	5 (3)	2 (2)	3 (3)		
One dose	4 (2)	2 (2)	2 (2)		
Two doses	29 (16)	14 (16)	15 (18)		
Three doses	139 (78)	73 (80)	66 (77)		
Vaccine hesitancy, mean (SD)	30.20 (4.84)	29.66 (5.31)	30.78 (4.24)	−1.544 (175)^b^	.12
Vaccine readiness, median (IQR)	2 (0-5)	1 (0-5)	2 (0-4.25)	0.601^d^	.55
Vaccine confidence, mean (SD)	11.69 (3.06)	11.48 (2.87)	11.91 (3.26)	−0.895 (175)^b^	.37
Vaccine-related health literacy, mean (SD)	10.64 (2.54)	10.74 (2.55)	10.55 (2.55)	0.495 (175)^b^	.62
Trust in government, mean (SD)	40.38 (26.8)	39.27 (27.48)	41.56 (26.17)	−0.565 (175)^b^	.57

a *t* test.

b Chi-square value.

c $ represents Hong Kong dollars (HK $). A currency exchange rate of HK $10,000=US $1273 is applicable.

d *z* score.

### Participant Characteristics

Table 1 describes participants’ characteristics at baseline. Among 177 participants, the mean age was 43.38 (SD 16.42) years, 65% (115/177) were female, 64.4% (114/177) had college or above education, 54.8% (97/177) were employed, and 68.4% (121/177) had a monthly household income above HK $20,000 (US $2548). The majority (152/177, 85.9%) of participants reported not having any chronic illnesses, and 50.2% (89/177) considered their health status as very good or good. In addition, 68.4% (121/177) had a history of testing positive for COVID-19, 75% (129/177) experienced side effects after receiving the COVID-19 vaccine, and 78.5% (139/177) had received 3 doses of COVID-19 vaccines. The sample showed moderate vaccine hesitancy (mean 30.20, SD 4.84; range 10‐50), low vaccine readiness (median 2 (0, 5), range 0‐10), moderate low trust in government (mean 40.38, SD 26.80; range 0‐100), and moderate vaccine-related health literacy (mean 10.64, SD 2.54; range 4‐16) at baseline. There are no significant group differences among all characteristics and outcomes at baseline.

### Changes in Vaccine Attitudes During the 5-Week Intervention and Participant Satisfaction

**Figure 3.**
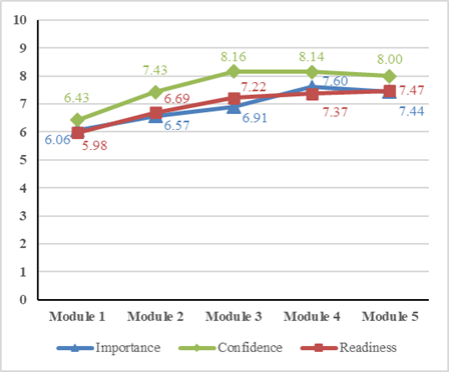
Changes in vaccine attitudes among participants in the intervention group.

Figure 3 presents weekly changes in vaccine importance, confidence, and readiness assessed by the chatbot among participants in the intervention group. During the 5-week intervention period, vaccine importance (from 6.06 to 7.44), confidence (from 6.43 to 8.00), and readiness (from 5.98 to 7.47) showed increasing trends. Participants in the intervention group reported moderate satisfaction with their participation and no significant group difference was identified when compared with the control group (mean 19.74, SD 0.91 vs mean 19.57, SD 0.96; *t*_141_=0.949; 2-tailed *P*=.34).

### Intervention Effectiveness

**Figure 4.**
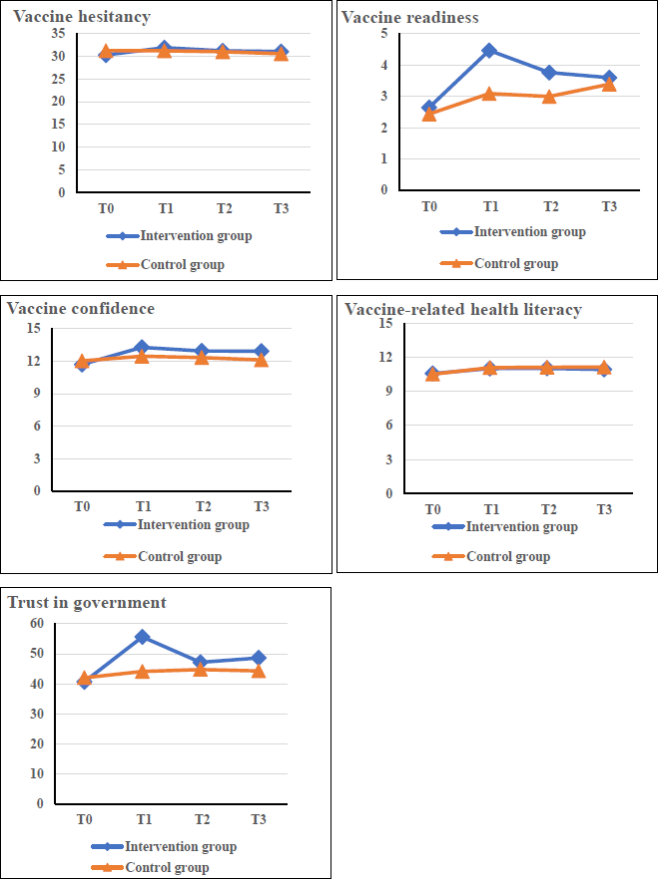
Changes in the scores of outcomes among 4 time points.

Table 2, Table 3, and Table 4 display the intervention effects between groups and Figure 4 provides graphical representations. For the primary outcome, decreases in vaccine hesitancy were observed in the intervention group at T1, T2, and T3. However, no significant time-group interaction was detected. For secondary outcomes, the intervention showed significant effects for time (*P*<.001), group (*P*=.03), and their interaction (*P*=.042) in vaccine readiness. A significant group difference at T1 was identified (*P*=.048) with a medium effect size (Cohen *d*=0.52, 95% CI 0.12-0.86) in favor of the intervention. The intervention also showed significant effects for time (*P*=.002), group (*P*=.04), and their interaction (*P*=.04) in trust in government. A significant group difference at T1 was identified (*P*=.049) with a medium effect size (Cohen *d*=0.54, 95% CI 0.20-0.88) in favor of the intervention. For vaccine confidence, significant effects were found for time (*P*<0.001), group (*P*=.048), and the interaction (*P*=.02). Although no significant group differences were observed in the post hoc comparisons, a small between-group effect size (Cohen *d*=0.39, 95% CI 0.05-0.72) was identified at T1 in favor of the intervention. Increases in vaccine-related health literacy were observed post the intervention and significant time effect was found (*P*=.01).

**Table 2.** Intervention effects by group assignment across time using generalized estimating equation.

Measures	T0^a^, mean (SE)	T1^b^, mean (SE)	T2^c^, mean (SE)	T3^d^, mean (SE)
Vaccine hesitancy				
Intervention group	30.27 (0.44)	31.86 (0.44)	31.17 (0.48)	31.03 (0.42)
Control group	31.23 (0.41)	31.18 (0.39)	30.97 (0.44)	30.53 (0.53)
Vaccine readiness				
Intervention group	2.65 (0.25)	4.47 (0.35)	3.76 (0.32)	3.60 (0.36)
Control group	2.44 (0.24)	3.09 (0.28)	3.00 (0.26)	3.39 (0.34)
Vaccine confidence				
Intervention group	11.71 (0.17)	13.29 (0.27)	12.95 (0.25)	12.94 (0.25)
Control group	12.04 (0.21)	12.45 (0.25)	12.34 (0.27)	12.12 (0.32)
Vaccine-related health literacy				
Intervention group	10.59 (0.13)	11.03 (0.22)	11.04 (0.20)	10.95 (0.19)
Control group	10.53 (0.13)	11.11 (0.19)	11.15 (0.21)	11.16 (0.22)
Trust in government				
Intervention group	40.68 (1.35)	55.64 (2.77)	47.19 (2.35)	48.64 (2.74)
Control group	42.03 (1.18)	44.10 (2.34)	44.77 (2.35)	44.34 (2.53)

a Baseline assessment.

b Post the intervention test.

c 3 months follow-up post the intervention.

d 6 months follow-up post the intervention.

**Table 3.** Tests of adjusted generalized estimating equation model effects.

Measures	Time effect		Group effect		Time-by-group effect	
	Wald Chi-square (*df*)	*P* value	Wald Chi-square (*df*)	*P* value	Wald Chi-square (*df*)	*P* value
Vaccine hesitancy	4.8 (3)	.18	0.07 (3)	.8	3.9 (1)	.27
Vaccine readiness	29.4 (3)	<.001	4.9 (3)	.03	8.2 (1)	.04
Vaccine confidence	25.6 (3)	<.001	3.9 (3)	.048	10.2 (1)	.02
Vaccine-related health literacy	11 (3)	.01	0.3 (3)	.59	0.6 (1)	.91
Trust in government	14.9 (3)	.002	4.4 (3)	.04	8.6 (1)	.04

**Table 4.** Between-group comparisons.

Measures		T1^a^, Mdiff (95% CI)	T2^b^, Mdiff (95% CI)	T3^c^, Mdiff (95% CI)
Vaccine hesitancy		0.682 (–1.092 to 2.456)	0.199 (–1.574 to 1.971)	0.509 (–1.412 to 2.430)
Vaccine readiness		1.377 (0.006 to 2.748)	0.760 (–0.422 to 1.942)	0.212 (–0.932 to 1.356)
*P* value^d^		0.048	0.603	0.957
ES^e^ (95%CI)		0.523 (0.188 to 0.863)	0.326 (–0.020 to 0.674)	0.074 (–0.272 to 0.421)
Vaccine confidence		0.835 (–0.289 to 1.960)	0.612 (–0.462 to 1.686)	0.829 (–0.356 to 2.013)
*P* value^d^		0.392	0.777	0.492
ES^e^ (95%CI)		0.385 (0.051, 0.721)	0.284 (–0.062 to 0.631)	0.345 (–0.002 to 0.700)
Vaccine-related health literacy		–0.084 (–0.897 to 0.729)	–0.111 (–0.914 to 0.692)	–0.210 (–1.062 to 0.642)
Trust in government		11.533 (0.032 to 23.033)	2.415 (–6.763 to 11.593)	4.299 (–6.409 to 15.008)
*P* value^d^		0.049	0.992	0.976
ES^e^ (95%CI)		0.539 (0.203 to 0.879)	0.126 (–0.219 to 0.471)	0.202 (–0.144 to 0.551)

a Post the intervention test.

b 3 months follow-up post the intervention.

c 6 months follow-up post the intervention.

d *P* value for the between-group difference measured at post-intervention, three-month, and six-month follow-up.

e Cohen *d* effect size (ES) was calculated using mean (SE) in the generalized estimating equation model for the between-groups effect.

### Qualitative results

Table 5 summarizes the characteristics of the 14 interviewees across 4 focus groups. In total, three categories were identified from content analysis of the interview data: (1) improved vaccine literacy, confidence, and trust in government; (2) hesitancy varied while readiness improved; and (3) facilitators, barriers, and recommendations of modifications on the intervention. Table 6 outlines the categories, subcategories, and example quotes.

**Table 5.** Focus group interviewee’s characteristics.

Case number	Sex	Age (y)	Changes on vaccine hesitancy (T1-T0)
Group 1 (75 min)			
G1P1	Male	22	1
G1P2	Female	21	6
G1P3	Female	24	−3
Group 2 (105 min)			
G2P1	Female	39	−3
G2P2	Male	33	0
G2P3	Male	35	−3
G2P4	Male	27	−11
Group 3 (80 min)			
G3P1	Female	32	−4
G3P2	Female	45	−5
G3P3	Female	49	−2
Group 4 (n=75)			
G4P1	Female	67	4
G4P2	Female	65	1
G4P3	Female	65	−6
G4P4	Male	66	−8

**Table 6.** Categories, subcategories, and example quotes.

Categories and subcategories	Example quotes
Improved vaccine literacy, confidence, and trust in government	
Increased literacy for seeking and understanding vaccine information	*Using the chatbot makes me think more deeply about my vaccine decision and prompts me to search online for more information before deciding.* [G1P2]
Improved confidence in vaccination	*The program provided me with rich information, relieved my concerns, and made me confident and knew about the vaccines I had.* [G3P1]
Improved trust in government-provided vaccine information	*I previously felt the vaccines were too fast produced and promoted by the government. This program provided data for its safety and side effects and made me feel reliable.* [G3P2]
Hesitancy varied while readiness improved	
Reduced hesitancy due to increased knowledge and confidence	*I am more accepting and less hesitant to take vaccines. I will not consider it if I do not receive the information and don’t know about these vaccines.* [G3P3]
Maintain hesitancy due to perceived low needs	*If the virus worsens and there are high death rates, I may reconsider getting vaccinated. For the current situation, I won’t consider getting vaccinated.* [G2P4]
Increased readiness due to enhanced literacy	*The program helps me decide which vaccines to take when I plan to take in the future.* [G3P2]
Facilitators, barriers, and recommendations of modifications on the intervention	
Perceived benefits, accessibility, and engaging design facilitated the use of the intervention	*I enjoy the multiple-choice questions in the module. Scoring well on them gives me a sense of achievement, making my participation meaningful.* [G2P4]
Infection peak passed, and technical limitations restrict the use of the intervention	*Timing was crucial. If the intervention was launched earlier, I would have been more proactive to use it. But at this point, the infection peak has passed. I’m less motivated to use it for vaccine information.* [G1P1]
Comprehensive content and technical refinement are recommended	*What I want now is not just news, but more functional features - like helping elderly users contact the hospital, make appointments, and report their conditions, instead of having to do it themselves.* [G2P2]

#### Category 1: Improved Vaccine Literacy, Confidence, and Trust in Government

##### Increased Literacy for Seeking and Understanding Vaccine Information

Participants acknowledged benefits from both the motivational chatbot and web-based educational modules with multimedia content, which facilitated vaccine information seeking and understanding.

*Using the chatbot makes me think more deeply about my vaccine decision and prompts me to search online for more information before deciding.*

[G1P2]

*The module uses both text and YouTube videos to clarify misconceptions and provide details on vaccine selection.*

[G3P2]

##### Improved Confidence in Vaccination

Participants indicated increased confidence in vaccines as they received rich information on vaccines following their participation in the intervention.

*The program provided me with rich information, relieved my concerns, and made me confident and knew about the vaccines I had.*

[G3P1]

*I did not have much confidence on vaccines as they were fast produced. This program provided clinic data and information, which made me clear about vaccine safety and increased my confidence.*

[G3P2]

##### Improved Trust in Government-Provided Vaccine Information

Participants suggested that the AI digital assistant provided solid data and engaging information, which helped them to understand and validate government-provided vaccine information.

*I previously felt the vaccines were too fast produced and promoted by the government. This program provided data for its safety and side effects and made me feel reliable.*

[G3P2]

*This program included information from the government website and presented information more elaborate and engaging, which helped me know about vaccines*.

[G3P1]

### Category 2: Hesitancy Varied While Readiness Improved

#### Reduced Hesitancy Due to Increased Knowledge and Confidence

Participants perceived a reduction in their vaccine hesitancy due to the AI digital assistant motivating them in information seeking and increasing their knowledge and confidence in vaccines.

*I am more accepting and less hesitant to take vaccines. I will not consider it if I don’t receive the information and don’t know about these vaccines*.

[G3P3]

*The program motivates me to reflect more clearly on vaccines and search for more information online before I consider whether to take vaccines or not*.

[G1P2]

#### Maintain Hesitant Due to Perceived Low Needs

Several participants maintained vaccine hesitancy, only considering vaccination if the external need becomes dire, such as the perceived threats to health, or the government making vaccination compulsory to access public places.

*If the virus worsens and there are high death rates, I may reconsider getting vaccinated. In the current situation, the infection cases have decreased much and the symptoms are not severe. I won’t consider getting vaccinated*.

[G2P4]

*I got vaccinated because it was compulsory. I will not consider vaccination unless it is required by the government for vaccination to access public places*.

[G1P2]

#### Increased Readiness Due to Enhanced Literacy

Participants indicated a better vaccine readiness, when required to vaccinate, due to increased health literacy following the use of the AI digital assistant.

*The program helps me decide which vaccines to take when I plan to take in the future*.

[G3P2]

*The program introduced comprehensive information and increased my understanding of vaccines. I will use this system to learn more about vaccines if it is compulsory to vaccinate*.

[G1P2]

### Category 3: Facilitators, Barriers, and Recommendations of Modifications on the Intervention

#### Perceived Benefits, Accessibility, and Engaging Design Facilitated the Use of the Intervention

Participants expressed positive feedback on the module, stating that its interactivity, accessibility, and informative design played a key role in facilitating the use and acceptance of the AI digital assistant tool.

*I enjoy the multiple-choice questions in the module. Scoring well on them gives me a sense of achievement, making my participation meaningful*.

[G2P4]

*I prefer this flexible program over lecture-style ones, as you don’t have to go somewhere to participate. It sounds very convenient*.

[G1P2]

*I’d rate it 8/10 - it cited different sources for comprehensive information and had a clear side effects table*.

[G1P3]

#### Infection Peak Passed and Technical Limitations Restrict the Use of the Intervention

Participants indicated that the timing of the AI digital assistant’s deployment impacted its usability and their motivation to engage with it. Also, they expressed that they faced technical limitations with the AI digital assistant and posed a barrier to the use of the digital tool.

*Timing was crucial. If the intervention was launched earlier, I would have been more proactive to use it. But at this point, the infection peak has passed. I’m less motivated to use it for vaccine information*.

[G1P1]

*I had an issue with a chatbot that I couldn’t click through. I entered and submitted the text, but it wouldn’t let me proceed. I had to click a few times over a few days*.

[G2P2]

*The public education category needs to be more comprehensive. For example, when the public seeks information about vaccines, they might associate it with public safety concerns. However, if the AI in this program goes beyond its predefined scope, it will be unable to respond*.

[G4P4]

#### Comprehensive Content and Technical Refinement Are Recommended

Participants provided feedback that the AI digital assistant could be improved through providing more comprehensive and practical functions, as well as technical refinements to streamline the conversational experience and reduce repetitiveness.

*What I want now is not just news, but more functional features - like helping elderly users contact the hospital, make appointments, and report their conditions, instead of having to do it themselves*.

[G2P2]

*It’s good to chat with the chatbot, but the conversations are too long and repetitive. It seems to be stuck in an infinite loop instead of finding the answers it wants, so I think it could be shortened*.

[G3P2]

*I believe that if this program continues to be updated and new units are released even after the completion of the initial five units, it will actually motivate us to use it more*.

[G4P4]

## Discussion

### Principal Findings

The study results suggest that integrating AI chatbot and MI techniques can benefit vaccine promotion by enhancing vaccine-related health literacy and cultivating positive attitudes to improve individuals’ readiness for vaccination. The findings underscore the potential of digital tools combined with communication techniques in public health strategies for coping with vaccine-preventable diseases more effectively.

The AI digital assistant did not significantly improve vaccine hesitancy, aligning with previous research that has shown mixed results for interventions such as virtual games, apps, and MI [41]. One possible explanation for insignificant changes is the deeply rooted nature of vaccine hesitancy, which may not be easily altered by short-term interventions [42]. Another potential explanation might be attributed to the intervention conducted postpandemic [43]. Our participants suggested in the interviews that the AI digital assistant is helpful in reducing hesitancy due to increased vaccine knowledge and confidence. However, they perceived a low necessity for vaccination due to the perceived low susceptibility and severity of infection post pandemic as well as the lack of compulsory policies. The findings implied both the internal and external factors should be considered for addressing vaccine hesitancy [34]. Specifically, joint efforts should be made by health professionals and health departments to increase individuals’ vaccine health literacy and confidence, improve their perceptions of susceptibility and severity, and introduce policies supportive of vaccination and acceptable to the general public [44]. In addition, the vaccine hesitancy scale was not specifically designed for COVID-19 vaccines but for general vaccines, possibly affecting the sensitivity of detecting changes in attitudes specific to the pandemic context [45]. Future research is recommended to use the measurement designed for specific vaccines and further explore the intervention effects.

The AI digital assistant achieved a significant improvement in vaccine readiness with a moderate effect size immediately post intervention [46]. This aligns with studies showing that tailored communication strategies, MI, and educational components can effectively boost short-term vaccine readiness through enhancing vaccine-related knowledge and confidence, though their long-term impact remains uncertain [41,47]. Qualitative feedback from our study indicated that participants perceived the information provided by the AI digital assistant facilitated their readiness for vaccination when they considered taking vaccines. Vaccine readiness was suggested to be directly associated with vaccination behavior. To preserve the initial gains in vaccine readiness for long-term effects, it should be beneficial to explore approaches such as “top-up” sessions, updating the intervention content to reflect current health concerns, or integrating reminders and prompts that reinforce the importance of vaccination over time [48]. While AI-driven digital tools have proven effective for short-term interventions, their long-term success likely depends on adapting content and delivery methods to better address evolving needs and maintain the motivation to vaccinate [49]. Interestingly, despite positive changes in vaccine readiness, vaccine hesitancy remained high in this study due to a low perceived vaccine necessity in the current postpandemic context. This finding revealed the complex nature of vaccination decision-making and the dynamic relationship between vaccine hesitancy and readiness over time. In addition, it is important to highlight that while the statistically significant improvement in vaccine readiness is noteworthy, it does not necessarily guarantee meaningful clinical changes in practical vaccination promotion [50]. Future implementation studies are needed to explore these findings further and address existing contextual barriers [51].

The intervention significantly boosted vaccine confidence from baseline. Although the intervention group maintained higher confidence, the between-group differences were not statistically significant, likely due to the small effect size and limited statistical power [52]. This aligns with studies on the impact of digital interventions on vaccine confidence, which suggest that both digital approaches combining educational content and educational interventions alone contribute to the increase in vaccine confidence [53,54]. Qualitative data indicated that the AI digital assistant’s tailored information and communicative features effectively motivated participants to seek information and addressed their concerns about vaccine safety, thereby enhancing vaccine confidence. Future research should consider maintaining these positive effects by developing interventions that continuously engage participants and address evolving concerns. In addition, the intervention led to a significant increase in trust in the government with a moderate effect size immediately post intervention, which could be attributed to a specific module content visualizing the government’s pandemic management efforts and validating government-provided vaccine information [55]. Evidence demonstrated that enhanced trust in government positively correlates with vaccine confidence and readiness, which may contribute to overall vaccine acceptance [13]. Personalized and empathetic communication, facilitated by chatbots’ real-time and tailored responses, played a crucial role in addressing individual concerns and building confidence in government initiatives [47]. This highlights the value of using the MI-oriented AI chatbot to deliver clear, comprehensive communication to maintain trust and enhance vaccine attitudes.

The AI digital assistant led to a slight and long-term increase in vaccine-related health literacy at postintervention and 3-month follow-ups. The slight decrease observed at the 6-month follow-up in our study could be attributed to the lack of ongoing reinforcement [56]. This finding is consistent with previous research, which has reported mixed results regarding the sustainability of vaccine-related health literacy. Some studies have shown lasting gains, while others have noted declines post intervention, potentially due to variations in the content, delivery methods, and the degree of participant engagement over time [57,58]. Qualitative feedback highlighted the AI digital assistant’s benefits in boosting vaccine health literacy, specifically with increased information-seeking motivated by the MI-oriented chatbot and improved understanding through the web-based educational modules. The findings suggest the application of emerging AI-powered conversational agents and MI techniques in health education for behavior change [59].

### Limitations

This study has several limitations that should be acknowledged. First, the reliance on self-reported measures and single-item measurements may have introduced response biases and measurement error, potentially affecting the reliability and validity of the data. The Adult Vaccine Hesitancy Scale used to measure vaccine hesitancy was designed for general vaccines, not specifically for COVID-19, which may have affected its sensitivity. The generalizability of the findings is also limited due to the study’s focus on a specific demographic—Hong Kong adults. Furthermore, the limited number of intervention sessions may have been insufficient to produce significant changes in vaccine health literacy and hesitancy, particularly concerning effect size. The relatively lower adherence rate in the intervention group compared with the control group suggests that maintaining long-term engagement with the digital vaccine intervention was a significant challenge. Although the AI-driven digital assistant demonstrated encouraging short-term effects, it proved challenging to maintain user engagement over a longer period, which may have an influence on long-term outcomes. In addition, the postpandemic environment likely influenced vaccine attitudes, as many participants perceived a lower necessity for vaccination due to the reduced perceived severity of COVID-19. This could have tempered the intervention’s effects on reducing participants’ vaccine hesitancy.

### Future Research Directions

The findings from this study have significant implications for public health strategies aimed at improving vaccine attitudes and coverage. Integrating MI techniques with AI-driven chatbots in health promotion efforts effectively addresses individual concerns and resistance about taking vaccines, enhancing vaccine readiness, and demonstrating their potential as practical tools for increasing vaccination rates [31]. Furthermore, qualitative feedback from participants suggests that refining the intervention content could further improve its efficacy. Specifically, incorporating more relevant and personalized information that addresses specific concerns could enhance the intervention engagement and long-term effectiveness [60]. Future studies should also examine the applicability of these digital interventions to other vaccine-preventable diseases and health behavior promotion, as well as their impact across diverse populations and settings [61,62]. Comparing the cost-effectiveness of AI-driven digital assistants with human-delivered MI interventions could provide valuable insights for optimizing resource allocation in public health initiatives. This broader perspective is essential for formulating more resilient and efficient ways to enhance vaccine attitudes and increase coverage on a wider scope. Our findings also suggest the importance of timely, attitude-targeted interventions in effectively shaping individuals’ acceptance of vaccines. Our program provides a foundation for making rapid adjustments to tailor the AI digital assistant for future emerging infectious diseases and newly developed vaccines.

### Conclusions

The theory- and evidence-based, MI-oriented AI digital assistant resulted in significant changes in vaccine readiness, while the effects on vaccine hesitancy were insignificant and remain to be examined in future research. Despite the notable immediate improvements in vaccine attitudes, the effects diminished over time, highlighting the necessity for continuous intervention engagement and refinement. The study findings underscore the potential of integrating MI-based communication techniques with AI technologies to enhance public health strategies aimed at promoting positive vaccine attitudes. This innovative approach offers a promising direction for future research and practice in health communication, emphasizing the need for accessible, sustainable, and tailored interventions to boost vaccine acceptance for coping with future pandemics.

## Supplementary material

10.2196/72637 Multimedia Appendix 1 Consent form.

10.2196/72637 Multimedia Appendix 2 Generalized estimating equation model results using complete case analysis.

10.2196/72637 Multimedia Appendix 3 Generalized estimating equation model results using complete case analysis.

10.2196/72637 Checklist 1 CONSORT-eHEALTH (Consolidated Standards of Reporting Trials of Electronic and Mobile Health Applications and Online Telehealth) checklist [63].

## Abbreviations

AI: artificial intelligence
CONSORT: Consolidated Standards of Reporting Trials
CONSORT-EHEALTH: Consolidated Standards of Reporting Trials of Electronic and Mobile Health Applications and Online Telehealth
MI: motivational interviewing
WHO: World Health Organization
