# Supplementary material for: Effects of a Theory- and Evidence-Based, Motivational Interviewing–Oriented Artificial Intelligence Digital Assistant on Vaccine Attitudes: A Randomized Controlled Trial
Source: J Med Internet Res. 2025 Aug 8;27:e72637. doi: 10.2196/72637 (PMC12334111; doi:10.2196/72637)
Supplement: Multimedia Appendix 1 [file jmir-v27-e72637-s001.pdf]

Consent form .

<u>參與研究同意書</u>
<u>CONSENT TO PARTICIPATE IN RESEARCH</u>

[智能疫苗助理]： 基於互聯網的心理健康教育協同人工智能數碼助理對降低香港人群接種 COVID-19疫苗猶豫程度的效果研究
[AI-driven Vaccine Communicator: The impact of a Web-based psychoeducation programme with a motivational AI-based digital assistant on Covid-19 vaccine hesitancy in Hong Kong's population

本人同意參與由李妍博士開展的上述研究。
I hereby consent to participate in the captioned research conducted by Dr. Li Yan.

本人知悉此研究所得的資料可能被用作日後的研究及發表，但本人的私隱權利將得以保留，即本人的個人資料不會被公開。
I understand that information obtained from this research may be used in future research and published. However, my right to privacy will be retained, i.e. my personal details will not be revealed.

研究人員已向本人清楚解釋列在所附資料卡上的研究程序，本人明瞭當中涉及的利益及風險；本人自願參與研究項目。
The procedure as set out in the attached information sheet has been fully explained. I understand the benefit and risks involved. My participation in the project is voluntary.

本人知悉本人有權就程序的任何部分提出疑問，並有權隨時退出而不受任何懲處。
I acknowledge that I have the right to question any part of the procedure and can withdraw at any time without penalty of any kind.

參與者姓名（全名）：
Name of participant (Full Name):

[redacted]

參與者簽署：
Signature of participant：

[redacted]

×

clear
