# Supplementary material for: Effects of a Theory- and Evidence-Based, Motivational Interviewing–Oriented Artificial Intelligence Digital Assistant on Vaccine Attitudes: A Randomized Controlled Trial
Source: J Med Internet Res. 2025 Aug 8;27:e72637. doi: 10.2196/72637 (PMC12334111; doi:10.2196/72637)
Supplement: Multimedia Appendix 2 [file jmir-v27-e72637-s002.docx]

Appendix. Module contents of AI-driven motivational digital assistant.

| Module | Website | AI chatbot |
| --- | --- | --- |
| Module 1: Basic Knowledge of COVID-19 | COVID-19 and variants  Symptoms and complications  Long-term effects  Vulnerable population  Mode of transmission  Viral infection detection methods | Explore the perceived severity  Explore the perceived susceptibility  Explore the perceived available health services |
| Module 2: Basic Knowledge of COVID-19 Vaccine | Vaccine popularization  Vaccine development process  Available vaccines in Hong Kong  Data-driven vaccine efficacy  Priority population and vaccination | Explore ambivalence to vaccination  Explore the pros and cons of vaccination  Evoking the importance of vaccination |
| Module 3: Common Questions about COVID-19 Vaccine | Q&A pre-vaccination like age limit, priority group, safety, and efficacy  Q&A at vaccination like dosage and mixed vaccination  Q&A post-vaccination like side effects and tips for medication | Identify information selection bias  Explore personal beliefs and their impact on vaccine decisions and health  Evoking the importance of vaccination |
| Module 4: Myths about COVID-19 Vaccines | Common myths/rumors regarding vaccine safety like plausible side effects  Common myths/rumors regarding vaccine efficacy | Identify myths and misinformation  Providing evidence-based information  Evoking informed vaccine decision making |
| Module 5: Efforts by the Hong Kong Government | Efforts by the Hong Kong government during the COVID-19 pandemic  Input of medical resources  Financial and other assistance  Humanistic care | Aware of the ongoing efforts to protect public health  Summarize the five modules and make an informed decision and plan |
