# Supplementary material for: Effects of a Theory- and Evidence-Based, Motivational Interviewing–Oriented Artificial Intelligence Digital Assistant on Vaccine Attitudes: A Randomized Controlled Trial
Source: J Med Internet Res. 2025 Aug 8;27:e72637. doi: 10.2196/72637 (PMC12334111; doi:10.2196/72637)
Supplement: Multimedia Appendix 3 [file jmir-v27-e72637-s003.docx]

Appendix. Generalized estimating equation model results using complete case analysis.

| Measures | Mean (SE) | | | | | Tests of adjusted GEE model effects | | | Between-Group Comparison | | |
| --- | --- | --- | --- | --- | --- | --- | --- | --- | --- | --- | --- |
|  | T0 ^a^ | T1^b^ | T2 ^c^ | | T3 ^d^ | Time effect | Group effect | Time-by-group effect | T1 ^b^ Mdiff | T2 ^c^ Mdiff | T3 ^d^ Mdiff |
|  |  |  | |  |  | Wald χ^2^ (*p*) | | | (95% CI) | (95% CI) | (95% CI) |
| **Vaccine hesitancy** |  |  | |  |  | 5.039 (0.169) | 0.055 (0.814) | 4.985 (0.173) | 0.827 (-1.264, 2.917) | 0.242 (-1.808, 2.291) | 0.546 (-1.575, 2.668) |
| Intervention group | 29.97 (0.41) | 31.77 (0.53) | | 31.14 (0.58) | 30.90 (0.51) |  |  |  |  |  |  |
| Control group | 31.12 (0.40) | 30.95 (0.44) | | 30.90 (0.46) | 30.36 (0.55) |  |  |  |  |  |  |
| **Vaccine readiness** |  |  | |  |  | 31.637 (**<0.001**) | 4.674 (**0.031**) | 8.432 (**0.038**) | 1.384 (0.022, 2.765) | 0.747 (-0.420, 1.914) | 0.252 (-0.947, 1.450) |
| Intervention group | 2.55 (0.26) | 4.48 (0.36) | | 3.74 (0.31) | 3.57 (0.36) |  |  |  |  |  |  |
| Control group | 2.43 (0.24) | 3.10 (0.28) | | 3.00 (0.25) | 3.32 (0.33) |  |  |  |  |  |  |
| *p ^e^* |  |  | |  |  |  |  |  | **0.049** | 0.597 | 0.981 |
| ES (95% CI) ^f^ |  |  | |  |  |  |  |  | 0.510 (0.177, 0.846) | 0.324 (-0.017, 0.668) | 0.090 (-0.257, 0.438) |
| **Vaccine confidence** |  |  | |  |  | 32.200 (**<0.001**) | 2.095 (0.148) | 11.938 (**0.008**) | 0.748 (-0.476, 1.972) | 0.655 (-0.499, 1.809) | 0.815 (-0.512, 2.142) |
| Intervention group | 11.48 (0.21) | 13.24 (0.32) | | 12.93 (0.28) | 12.82 (0.30) |  |  |  |  |  |  |
| Control group | 11.97 (0.24) | 12.50 (0.26) | | 12.27 (0.28) | 12.01 (0.34) |  |  |  |  |  |  |
| *p ^e^* |  |  | |  |  |  |  |  | 0.392 | 0.777 | 0.492 |
| ES (95% CI) ^f^ |  |  | |  |  |  |  |  | 0.301 (-0.028, 0.632) | 0.287 (-0.057, 0.633) | 0.306 (-0.039, 0.653) |
| **Vaccine-related health literacy** | |  | |  |  | 9.065 (**0.028**) | 0.204 (0.651) | 0.705 (0.872) | -0.006 (-0.838, 0.825) | 0.149 (-0.777, 1.076) | 0.080 (-0.865, 1.025) |
| Intervention group | 10.70 (0.29) | 11.06 (0.26) | | 11.17 (0.25) | 11.01 (0.29) |  |  |  |  |  |  |
| Control group | 10.42 (0.26) | 11.06 (0.23) | | 11.02 (0.21) | 10.93 (0.25) |  |  |  |  |  |  |
| **Trust in government** |  |  | |  |  | 17.177 (**0.001**) | 4.093 (**0.043**) | 9.891 (**0.020**) | 12.119 (0.031, 23.925) | 3.142 (-6.634, 12.918) | 4.041 (-7.255, 15.337) |
| Intervention group | 39.86 (1.21) | 55.82 (2.92) | | 47.39 (2.45) | 48.52 (2.97) |  |  |  |  |  |  |
| Control group | 41.54 (0.96) | 43.70 (2.39) | | 44.25 (2.49) | 44.48 (2.69) |  |  |  |  |  |  |
| *p ^e^* |  |  | |  |  |  |  |  | **0.038** | 0.982 | 0.982 |
| ES (95% CI) ^f^ |  |  | |  |  |  |  |  | 0.538 (0.206, 0.873) | 0.154 (-0.186, 0.495) | 0.176 (-0.168, 0.521) |

Note. Wald χ2 = Wald Chi-square, p = p-value; GEE = Generalized estimating equation; SE = standard error.

a Baseline assessment; b Post the intervention test; c Three months follow-up post the intervention; d Six months follow-up post the intervention; e p Value for the between-group difference measured at post-intervention, three-month, and six-month follow-up; f Cohen’s d ES was calculated using mean (SE) in the GEE model for the between-groups effect
